# Analysis of the Functional Outcome of Arthroscopic Anterior Cruciate Ligament Reconstruction Using the Central Quadriceps Tendon Graft

**DOI:** 10.7759/cureus.65351

**Published:** 2024-07-25

**Authors:** Ambareesh Parameshwar, Lanka Bhupati Kumar, Supreeth R Donthi, S Gurucharan, Nishanth J Reddy, Varun GBS, Vishwanath M S, Amruta Gurudatta, Mohammed Shahid

**Affiliations:** 1 Trauma and Orthopedics, Vydehi Institute of Medical Sciences and Research Centre, Bangalore, IND; 2 College of Medicine, Vydehi Institute of Medical Sciences and Research Centre, Bangalore, IND; 3 Orthopedics, Vydehi Institute of Medical Sciences and Research Centre, Bangalore, IND

**Keywords:** sports injuries, knee stability, arthroscopy, tegner lysholm knee score, quadriceps tendon graft, acl reconstruction, anterior cruciate ligament

## Abstract

Introduction: Anterior cruciate ligament (ACL) injuries are common, particularly among athletes, and often result in knee instability and decreased functionality. Arthroscopic ACL reconstruction is the standard treatment, typically using a patellar tendon bone graft (PTBG) or hamstring tendon graft (HTG). The central quadriceps tendon graft (QTG) has been proposed as a superior alternative due to its structural properties.

Methodology: This study involved patients undergoing ACL reconstruction using the central quadriceps tendon graft. Functional outcomes were assessed using the Tegner Lysholm knee score were assessed at preoperative and postoperative intervals of two weeks, three months, and six months. Statistical analysis compared these scores over time.

Results: Among the subjects, 90.6% were male and 9.4% were female. Injuries primarily resulted from sports activities and road traffic accidents (46.9% each). Right-side injuries were more prevalent (65.6%). The mean time from injury to surgery was 9.37 months. The mean graft size was 8.75 mm, and the mean tourniquet time was 105.94 minutes. Preoperative tests showed positive results for anterior drawer, Lachman, and pivot shift tests in most patients, which were negative postoperatively. Significant improvements in knee flexion and Lysholm knee scores were observed. Preoperative knee flexion ranged from 0-100° to 0-120°, improving to 0-120° to 0-130° six months postoperatively. The mean Lysholm knee score improved from 47.06 preoperatively to 93.16 at six months. Excellent outcomes were seen in 78.1% of the patients, with 21.9% achieving good outcomes.

Conclusion: The central quadriceps tendon graft is an effective option for ACL reconstruction, offering excellent functional outcomes and low complication rates. It shows promise as a better alternative to traditional graft types, although further research is necessary to confirm these findings.

## Introduction

The anterior cruciate ligament (ACL) is a critical structure in the knee joint, responsible for stabilizing the knee by preventing the anterior translation of the tibia relative to the femur. Injuries to the ACL are common, particularly among athletes, and can lead to significant functional impairment, pain, and instability of the knee [1]. The severity of these symptoms often depends on the nature of the trauma, the level of impact, and other factors, such as age, gender, and pre-existing conditions, like osteoarthritis [2-6]​​.

Arthroscopic ACL reconstruction has become the gold standard for treating ACL injuries. Traditionally, the reconstruction has utilized grafts such as the patellar tendon bone graft (PTBG) or the hamstring tendon graft (HTG). However, the central quadriceps tendon graft (QTG) has recently been explored as an alternative due to its superior biomechanical properties. The quadriceps tendon is longer and wider and has approximately 50% more mass than the BPTB tendon, offering greater tensile strength, which may translate to better stability and functional outcomes for patients​​ [7,8].

Despite the availability of various graft options, primary ACL reconstruction still faces a failure rate of up to 5% [3]. This highlights the need for improved graft materials and techniques. Recent studies have suggested that the central quadriceps tendon proximal to the patella could be a reliable graft with better outcomes compared to other graft types [7]. The present study aims to evaluate the functional outcomes of ACL reconstruction using the central quadriceps tendon graft, assessed by the Tegner Lysholm knee score, to determine its efficacy and potential benefits over traditional graft options.

## Materials and methods

The study was conducted in the Department of Orthopaedics at Vydehi Institute of Medical Sciences and Research Centre, Bangalore, after approval from the Institutional Ethics Review Board (IERB) (no. VIEC/PG/APP/031/2020-21). The study included patients of either gender with ACL tears undergoing ACL reconstruction. This research was a descriptive study conducted over 18 months, from January 2021 to June 2022. A total sample size of 32 patients was determined using the formula for proportions.

Inclusion criteria

Patients with primary ACL injury, ACL injury with or without meniscal injury, and no previous knee surgeries were included.

Exclusion criteria

Patients with systemic infection or sepsis, additional ligamentous laxity in the affected knee, active articular infection or inflammatory joint disease, multiple ligament injuries, partial- or full-thickness cartilage defects, and femoral condyle fixation with an interference screw were excluded.

The study involved 32 patients with complete ACL tears who underwent arthroscopic ACL reconstruction using central quadriceps tendon grafts. Comprehensive case histories, including sociodemographic data and other medical conditions, were recorded. Preoperative preparation included X-ray and MRI imaging. Preoperative strength and range of movement of the knee joint were measured and documented. Static and dynamic quadriceps exercises were taught to patients while awaiting surgery. All patients were enlightened on the standard postoperative rehabilitation. Patients then underwent surgery according to the described methodology, and postoperative evaluations were conducted using X-rays and the Tegner Lysholm knee score to assess functional outcomes. Data were entered into a Microsoft Excel data sheet (Microsoft Corporation, United States) and analysed using IBM SPSS Statistics for Windows, Version 22.0 (released 2013, IBM Corp., Armonk, NY). A p-value of <0.05 was considered statistically significant after assuming all the rules of statistical tests.

## Results

The study analysed 32 patients who underwent ACL reconstruction using central quadriceps tendon grafts. The demographic and clinical characteristics of the study population are summarized in Table 1. In the study, 62.5% were in the age group <30 years, 25.0% were in the age group 31 to 40 years, and 12.5% were in the age group >40 years. Most (90.6%) were males and 9.4% were females. The most common mode of injury was sports injury and road traffic accidents (RTA) accounting for 46.9% of the subjects, while only 6.2% of the subjects reported falling from height. More than half (65.6%) had right-side injuries and 34.4% had left-side injuries.

**Table 1:** General profile of the subjects

Demographic characteristics		Count	%
Age	<30 years	20	62.5%
	31 to 40 years	8	25.0%
	>40 years	4	12.5%
Gender	Female	3	9.4%
	Male	29	90.6%
Mechanism of injury	Sports	15	46.9%
	RTA	15	46.9%
	Falling from height	Falling from height	Falling from height
Side of injury	Right	21	65.6%
	Left	11	34.4%

The mean time of injury to surgery was 9.37 ± 14.379 months, the mean size of the graft was 8.75 ± 0.762 mm, and the mean tourniquet time was 105.94 ± 10.03 minutes (Table 2).

**Table 2:** Distribution of parameters

Parameter	Time of injury to surgery (months)	Size of graft	Tourniquet time in minutes
N	32	32	32
Mean	9.37	8.750	105.94
SD	14.37	0.762	10.035
Median	4.00	9.000	105.00

**Figure 1:**
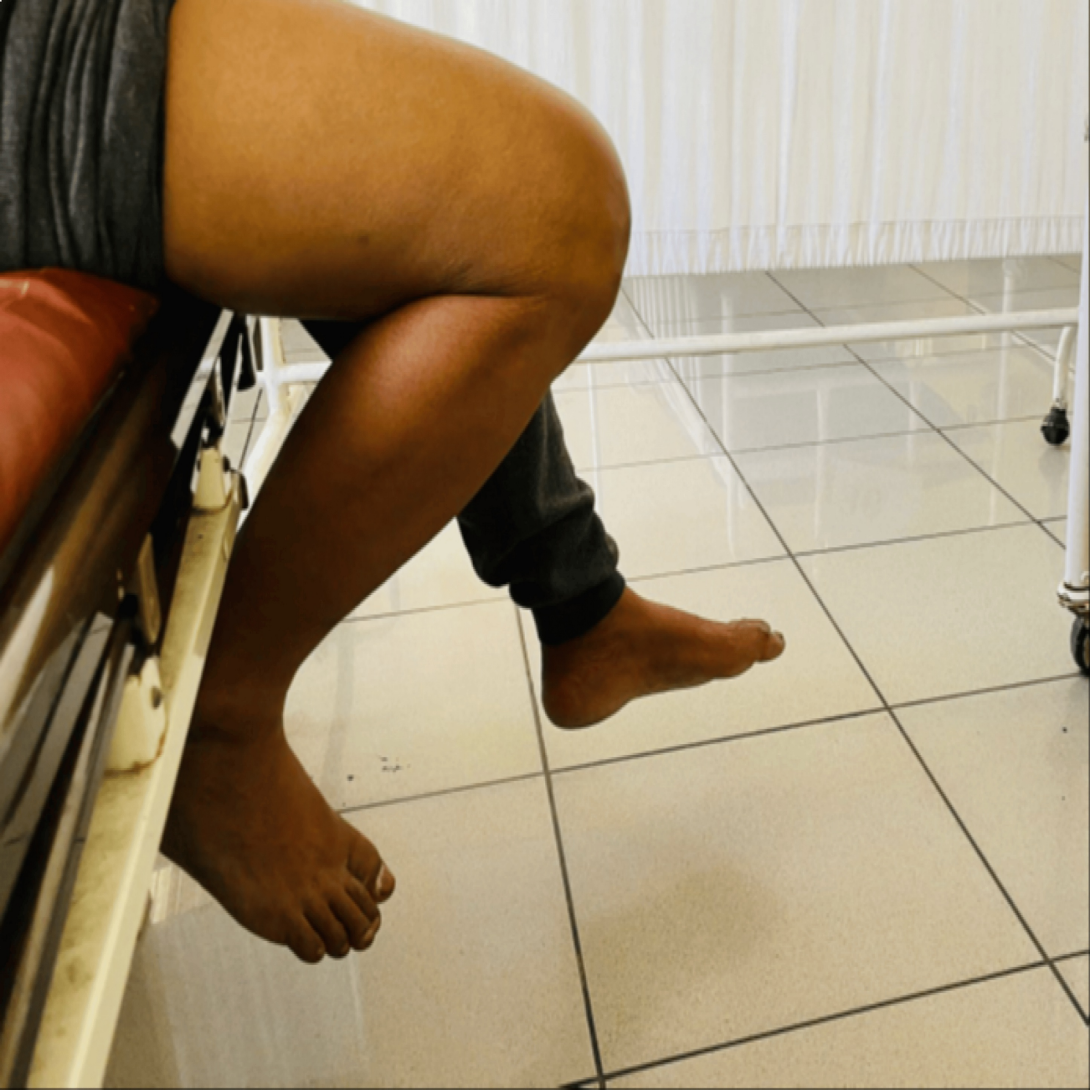
Case 1: six-month follow-up knee flexion

**Figure 2:**
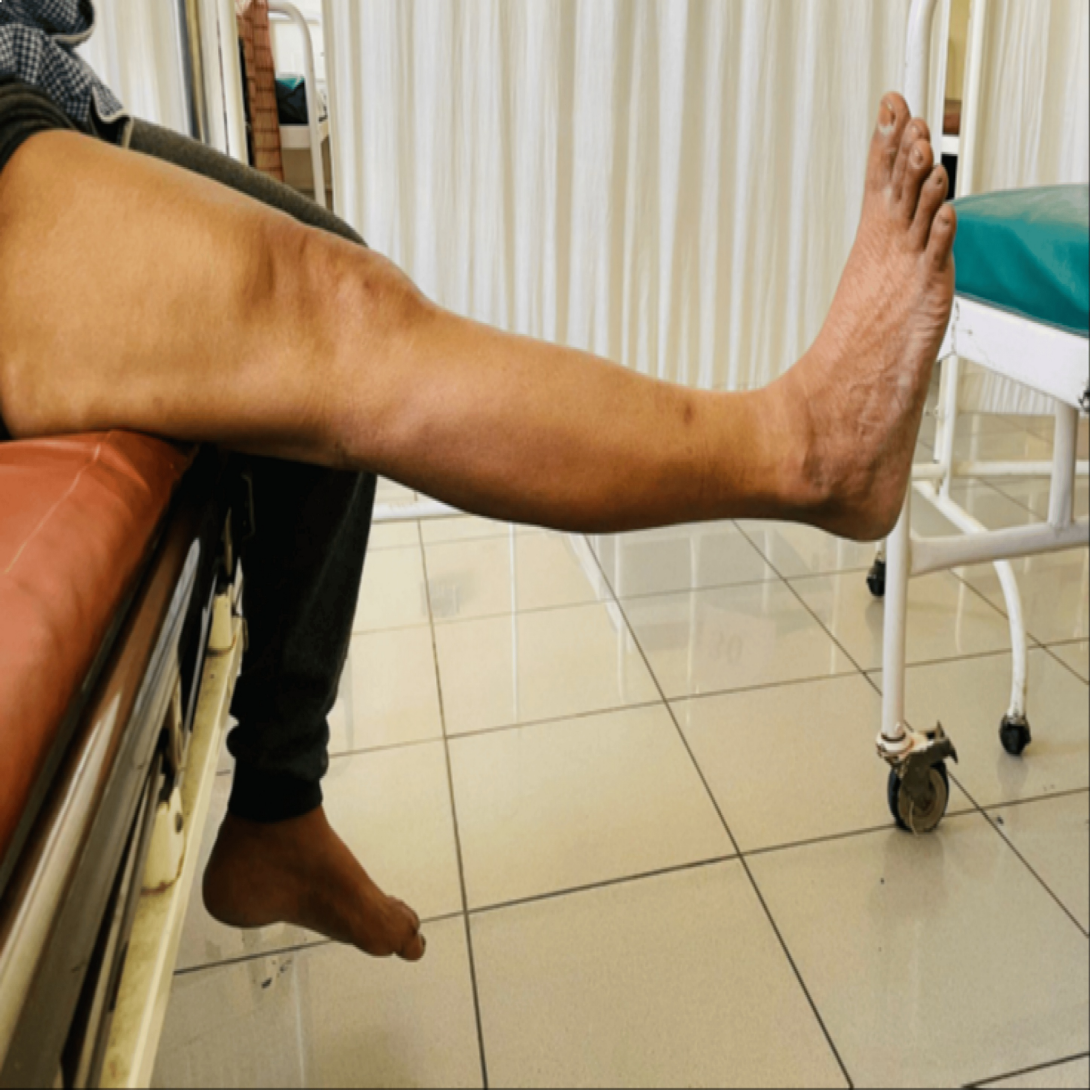
Case 1: six-month follow-up knee extension

**Figure 3:**
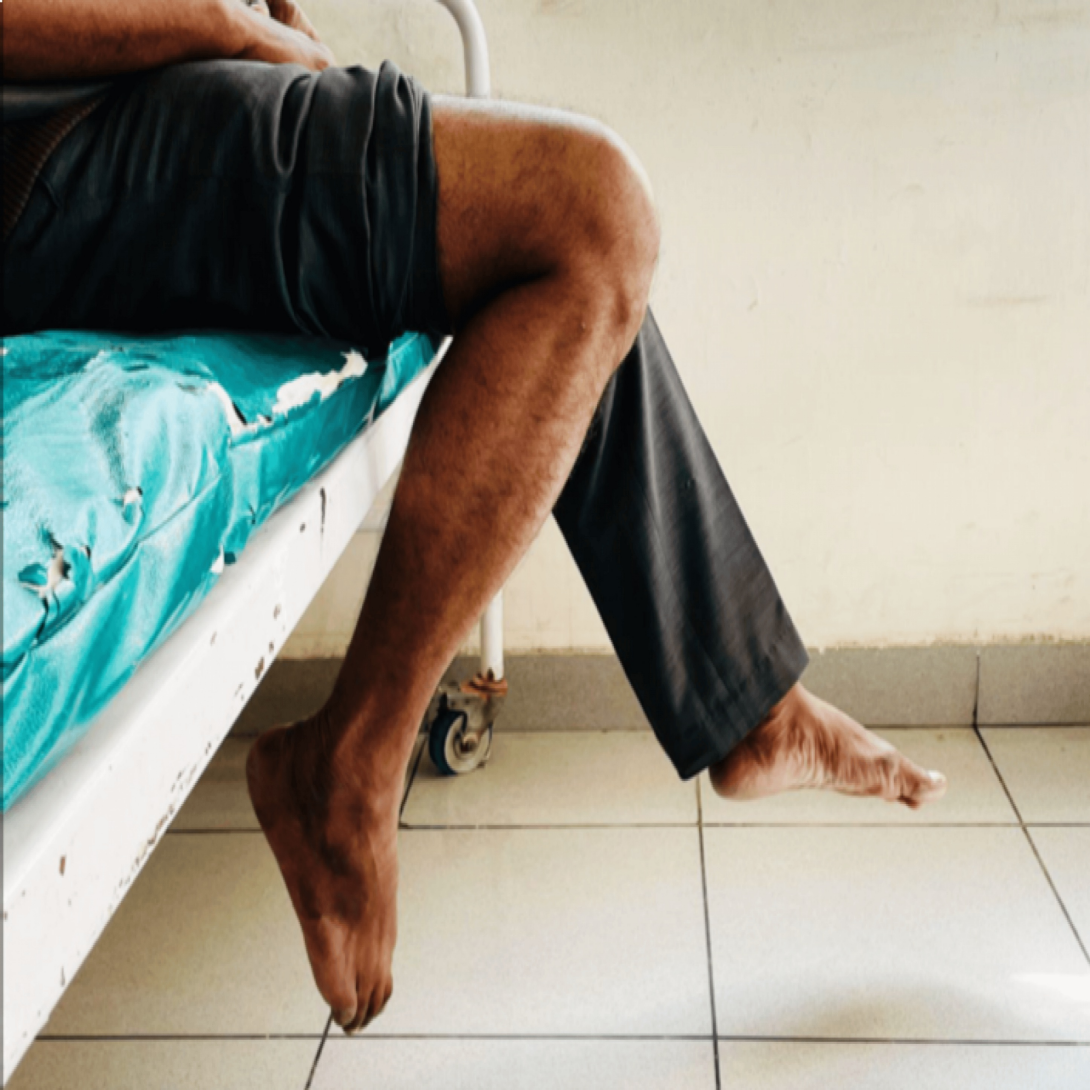
Case 2: six-month follow-up knee flexion

**Figure 4:**
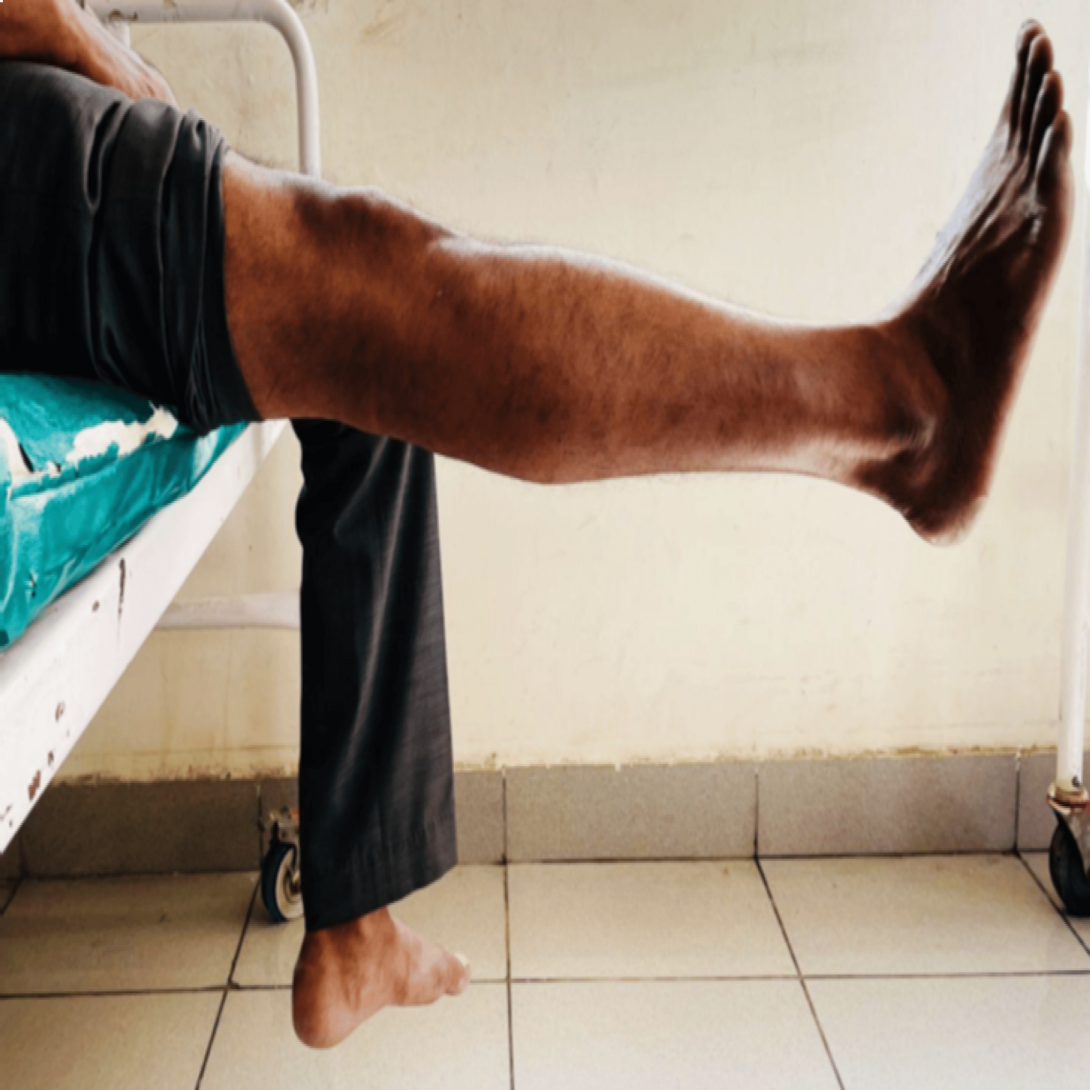
Case 2: six-month follow-up knee extension

Preoperatively, the anterior drawer test was positive in 87.5% of the patients, while the Lachman and pivot shift tests were positive in 100% of the patients. Postoperatively, all three tests were found to be negative (Table 3). Figure 1 and Figure 2 show the knee ROM of one patient (case 1), and Figure 3 and Figure 4 show the knee ROM of another patient (case 2) at the six-month follow-up.

**Table 3:** Knee test findings at pre-op and post-op

Test performed	Result	Count (n = 32)	%
Anterior drawer test	Negative	4	12.5%
	Positive	28	87.5%
Post-op anterior drawer test	Negative	32	100.0%
Lachman test	Positive	32	100.0%
Post-op Lachman test	Negative	32	100.0%
Pivot shift test	Positive	32	100.0%
Post-op pivot shift test	Negative	32	100.0%
Knee flexion pre-op	0-100	9	28.1%
	0-110	17	53.1%
	0-120	6	18.8%
Knee flexion at six months	0-120	17	53.1%
	0-130	15	46.9%

The mean Tegner Lysholm knee score at pre-op was 47.06 ± 7.26, 68.59 ± 4.31 at two weeks, 85.61 ± 5.28 at three months, and 93.16 ± 2.52 at six months. There was a significant increase in the Tegner Lysholm knee score at two weeks, three months, and six months compared to the pre-op value (Table 4).​​

**Table 4:** Tegner Lysholm knee score at different periods of follow-up

Tegner Lysholm knee score	Mean	SD	Median	P-value
Pre-op	47.06	7.26	48	
Post-op at two weeks	68.59	4.31	70	<0.001
Post-op at three months	85.61	5.28	87	<0.001
Post-op at six months	93.16	2.52	94	<0.001

In this study, 78.1% had excellent grades and 21.9% had good outcomes (Table 5)​.

**Table 5:** Grade distribution

Grade	Count	%
Excellent	25	78.1%
Good	7	21.9%

In this study, 3.1% of the subjects had pain after one year and another 3.1% had extension lags, whereas 93.8% had no complications (Table 6)​.

**Table 6:** Complication distribution

Complications	Count	%
Nil	30	93.8%
Pain after one year	1	3.1%
Extension lag	1	3.1%

The results of this study were compared with the existing literature. Sharma et al. reported similar improvements in Lysholm scores over time, with their mean scores at pre-op, three months, six months, and one year being 44.34, 78.98, 87.86, and 91.58, respectively​​ [9]. Runer et al. found mean preoperative and 24-month follow-up Lysholm scores of 93.1 and 86.0, respectively [10], while Galan et al. reported scores of 64 and 91.0 over a five-year follow-up period​​ [11]. Overall, the central quadriceps tendon graft was found to be an effective alternative for ACL reconstruction, with good to excellent outcomes and low complication rates​​.

## Discussion

Despite advances in surgical techniques and the use of various graft types, primary ACL reconstruction failure rates remain below 5% [3]. Among the graft options, bone-patellar tendon-bone (BPTB) autografts are favoured due to their effectiveness and durability compared to allografts, which can be costlier and less durable ​[3,8]​. This study focused on the functional outcomes of ACL reconstruction using a central quadriceps tendon graft. The quadriceps tendon graft offers a promising alternative due to its high properties in restoring knee stability and muscle strength. The functional outcomes were measured using the Tegner Lysholm knee score at various follow-up intervals.

There was a significant improvement in the Tegner Lysholm knee scores from the preoperative (mean 47.06) to postoperative periods at two weeks (mean 68.59), three months (mean 85.61), and six months (mean 93.16), indicating substantial functional recovery (p < 0.001)​​. At the six-month follow-up, 78.1% of the patients achieved an "excellent" outcome, and 21.9% had a "good" outcome, demonstrating the procedure's overall success​​. The study reported minimal complications, with 93.8% of patients experiencing no complications, 3.1% reporting pain after one year, and 3.1% having an extension lag​​. The mean size of the central quadriceps tendon graft used in our study was found to be 8.75 mm, with a standard deviation of 0.762 mm, which was found to be sufficient for ACL reconstruction.

This study's results align with other research indicating the effectiveness of quadriceps tendon grafts in ACL reconstruction. Studies have shown that quadriceps tendon grafts provide excellent biomechanical strength and are associated with low donor-site morbidity. In addition, the graft's size and volume can be customized to the patient's needs, offering a versatile option for surgeons​​ [8,9,12]. The findings suggest that the central quadriceps tendon graft is a viable and effective option for ACL reconstruction, providing significant functional improvements and a low complication rate. This supports its use as an alternative to traditional grafts, particularly in patients where other graft options may not be suitable.

## Conclusions

The study demonstrates that arthroscopic ACL reconstruction using the central quadriceps tendon graft provides excellent functional outcomes, as evidenced by significant improvements in the Tegner Lysholm knee scores over six months postoperatively. With a high rate of excellent and good outcomes and minimal complications, the central quadriceps tendon graft proves to be a viable and effective alternative to traditional graft options. These findings support its use in clinical practice, offering a promising option for restoring knee stability and function in patients with ACL injuries. Further research with larger sample sizes and longer follow-up periods is recommended to confirm these results and optimize surgical techniques and rehabilitation protocols.
